# P01-12 The Netherlands united by sport: implementtion and monitoring of the national sports agreement in 2019

**DOI:** 10.1093/eurpub/ckac095.012

**Published:** 2022-08-29

**Authors:** Wanda Wendel-Vos, Lilian van den Berg, Hugo van der Poel

**Affiliations:** 1 Centre for Nutrition, Prevention and Health Services, National Institute for Public Health and the Environment, Bilthoven, The Netherlands; 2 Knowledge Centre for Sport & Physical Activity Netherlands, Ede, The Netherlands; 3 Mulier Institute, Utrecht, The Netherlands

**Keywords:** National Policy, Implementation, Monitoring

## Abstract

**Issue/problem:**

The favorable international position of the Netherlands in terms of sport an physical activity is no reason for those involved in policy to lean back. The motor skills of our children are decreasing, some population groups never engage in sports, respectful behavior in sports needs our attention and the traditional sports clubs are under pressure due to declining numbers of members and volunteers. To tackle these issues, for the first time in history, the Netherlands have a National Sports Agreement (NSA) involving a numerous set of stakeholders besides the national government.

**Description of the problem:**

Through the NSA, we want to make sport enjoyable for everyone. Now and in the future, without any restrictions and in a safe and healthy environment. The sports infrastructure will be strengthened at every level: locally, regionally and nationally. Policy makers want to involve communities of practice and knowledge from monitoring and science in order to be able to adjust policy on the short term and create a ‘self-learning' policy process.

**Results:**

Local and regional stakeholders were invited to strengthen and start collaboration and compose local and regional sports agreements in line with the NSA which has six main ambitions: inclusive sport, sustainable sports facilities, vital providers, positive sports culture, enjoying exercise from an early age and elite sport inspires. As registered on 08-11-2019, 339 out of the 355 municipalities in the Netherlands have started work on a local sports agreement. Thirty nine local agreements were already in place covering 45 municipalities. From the national level, vouchers are made available for local stakeholders to facilitate implementation of particular interventions within a certain ambition on the local level. A consortium of national knowledge institutes provided local policy makers with training sessions facilitating them to incorporate local data, facts and figures in the local sports agreements. Twice a year, National Parliament is informed about the efforts taken and results obtained. On a continuous basis, for every ambition, a set of indicators is disseminated through the website www.sportenbewegenincijfers.nl

